# Stalking Wild Maize: Taxonomy, Plant Exploration, and the Search for Corn’s Origins in South America

**DOI:** 10.1162/jinh_a_02001

**Published:** 2024-04-19

**Authors:** Helen Anne Curry

**Affiliations:** Georgia Institue of Technology

**Keywords:** plant exploration, taxonomy, evolution, ethnobotany, maize

## Abstract

In the twentieth century, plant explorers in many countries were enlisted in the assembly of seed and plant collections that brought together hundreds and sometimes thousands of varieties of the same crop species. These collections were, and are, understood chiefly as the foundations for effective plant breeding. However, like other biological collections, crop seed collections were also essential tools of taxonomy: their study was both conditioned by and productive of evolutionary narratives about plant cultures and human natures. This crop taxonomic enterprise has been subject to far less scrutiny than its agronomic counterpart. In this article, I redress that imbalance through an account of a search for *Zea mays* (maize or corn) and its wild relatives in South America in the 1940s. As I show, developing taxonomies of cultivated plant species, and especially accounting for distinct local forms of these crop plants, was a profoundly interdisciplinary enterprise. It was also a project conditioned by researchers’ expectations of the places, peoples, and plants they would encounter. The taxonomy of maize, like other taxonomic enterprises, emerged as a mirror of those who taxonomized.

On 6 October 1941 the ethnobotanist Hugh Cutler and his wife Marian set sail from New York City on the *S.S. Uruguay*, a passenger ship bound for Rio de Janeiro. The vicissitudes of the war in Europe pressed upon its travelers. Everyone on board was there “by necessity” according to Marian, including a corps of sailors bound for war work on whaling ships. Even with Rio-bound entertainers including a “little Mexican singer of the spitfire type” and the “bearded Boa Montana, 325 pound wrestler” among their fellow second-class passengers, the journey wasn’t as lively as the Cutlers would have liked. No wonder, with rumors pinging around the ship that the cargo included lend-lease airplane engines and the crew’s lookouts on high alert for any suspicious boats sharing the seas. A few months later, after the bombing of Pearl Harbor, the *Uruguay* would be requisitioned for U.S. military transport.^1^

What necessity had induced the Cutlers to make the journey to Brazil in the midst of war? It was not missionary zeal, nor industrial opportunity, nor economic need, as with their fellow passengers. It was a taxonomic puzzle. Hugh had been hired by the botanist Paul Mangelsdorf of Harvard University to collect samples of *Zea mays*, also known as domesticated maize or corn, and its wild relatives. If carefully preserved, labelled, and shipped, these exemplars could reveal corn’s South American history to Mangelsdorf from the relative comfort of the Harvard Botanical Museum. To this demanding yet straightforward task Mangelsdorf added an ambitious request. He asked Hugh to locate an as-yet-unknown “wild maize” that he predicted would be growing in the lowlands of southwestern Brazil, northeastern Bolivia, or Paraguay. This find, if found, would shore up Mangelsdorf’s unorthodox claims about the evolutionary origins of *Zea mays*. A trophy ear would feature centrally in a display of North, Central, and South American maize varieties that he planned to create at the Botanical Museum to illustrate the evolution and diversification of the species. Better still, it would position him at the unquestioned center of research on the hemisphere’s most important crop.^2^

In the twentieth century, plant explorers like Cutler were enlisted by state agricultural programs in many countries to assemble seed and plant collections that brought together hundreds and sometimes thousands of local varieties of crop species and often samples of their wild relatives as well. These collections were, and are, understood as the foundations for effective plant breeding, stockpiles of “genetic resources” to be mined for traits that will keep agricultural productivity ticking upward. Historians’ accounts show them to have been prized as national resources, taken as spoils of war, and subject to international treaties. But crop seed collections were, like other biological collections, also essential tools of taxonomy: their study was both conditioned by and productive of evolutionary narratives about plant cultures and human natures. This taxonomic enterprise, in which researchers like Mangelsdorf used collections of farmers’ varieties and crop wild relatives assembled through sometimes wide-ranging exploration missions to map the evolutionary origins and diversification of food crops, has been subject to far less historical scrutiny than its production-oriented agronomic counterpart.^3^

This article seeks to redress that imbalance through an account of Hugh and Marian Cutler’s exploration of maize in South America. Although the Cutlers intended to be abroad for just one year, the entry of the United States into the war and their openness to unplanned adventures kept them in South America until 1945. Their search for samples of *Zea mays* and its wild relatives and evidence of Mangelsdorf’s projected wild *Zea* ancestor carried them down rivers in dugout canoes, across borders on trains and planes, and into the hills on horseback. It connected them to diverse maize experts in South America, as they learned from local growers and set up shop in new colleagues’ labs or multiplied seed collections in their fields. It strengthened links back home as their examples of farmers’ maize varieties and *Zea* relatives sustained desk research in Massachusetts, Missouri, and beyond. Their eventual aggregation of samples was, according to Mangelsdorf, “the most comprehensive and valuable collection of South American maize which has even been made.” Still, the ancestral wild maize remained elusive.^4^

The Cutlers’ travels generated a rich documentary trail of letters, diaries, photographs, and eventual publications. These materials show that developing taxonomies of cultivated plant species, and especially accounting for distinct local forms of these crops, was a profoundly interdisciplinary enterprise. The methods and assumptions of botany, genetics, anthropology, and archaeology intersected with the knowledge and skills of breeding, farming, cooking, and pharmacy. Reconstructing the Cutlers’ journey and related research on maize further reveals crop taxonomy as a project conditioned by researchers’ expectations of the places, peoples, and plants they would encounter. The taxonomy of maize, like other taxonomic enterprises, emerged as a mirror of those who taxonomized. My account of the ways that researchers like Paul Mangelsdorf and Hugh Cutler pursued maize plants and knowledge about them ultimately underscores the diverse purposes that collecting and taxonomizing domesticated crops and their relatives could serve—offering a reminder that agricultural development was only one of many agendas inscribed into twentieth-century crop plant exploration.^5^

## The search for corn’s origins

Although they set off from New York City harbor, the origin of the Cutlers’ journey to South America lay in a Texas cornfield. In 1927, Paul Mangelsdorf, then an agronomist and breeder at the Texas Agricultural Experiment Station in College Station, paid a visit to colleagues at a site near the Gulf Coast. As he toured their fields, he encountered gamagrass, a tall, perennial grass of the genus *Tripsacum*, growing in abundance in the railroad right of way that cut through the station. Mangelsdorf had seen examples of *Tripsacum* the previous year while immersed in maize genetics as a student at Harvard University and been struck by the plants’ similarities to maize. He’d wondered then: Had botanists placed the genus *Tripsacum* too far from *Zea mays* on their evolutionary tree? In Texas, the sight of *Tripsacum* growing in close proximity to maize raised the question again. He soon set out to resolve these evolutionary concerns, with botanical knowledge and cytogenetic studies as his go-to tools.^6^

Unlike many domesticated crops, corn had no obvious immediate wild ancestor. Figuring out where the dominant crop of the Americas had originated therefore hinged on understanding its nearest relatives. In the 1920s, botanists agreed that these relatives included the six or seven grasses classed as *Tripsacum* as well as two weedy grass species from Mexico and Guatemala known as teosinte, then placed in the genus *Euchlaena*. In theorizing the origins of maize, teosinte gathered the lion’s share of attention. Teosintes had the same number of chromosomes as *Zea mays* and hybrids of the two produced viable seeds. This compatibility led some botanists to conclude that teosinte and maize should be placed in the same genus (as they are today) and to suggest teosinte as maize’s wild ancestor. Not everyone was convinced, however, and often for an obvious reason. The female flowers of teosinte and the seed spike that results from them look nothing like their counterparts in maize. It was difficult to imagine how teosinte’s tiny spike of six to twelve small seeds could have transformed into the familiar immense ear of the maize plant with its hundreds of plump kernels. An intermediate form that bridged the gulf between these two might have made the evolutionary relationship more plausible, but no such plant had been found. As a result, other hypotheses continued to hold sway, such as the idea that maize, teosinte, and *Tripsacum* had all descended from a remote common ancestor.^7^

After seeing gamagrass growing wild in Texas, Mangelsdorf turned his research attention to the several *Tripsacum* species, hoping for new insights into their relationship to maize. He made experimental crosses of teosinte and *Zea* as well as *Tripsacum* and *Zea* and invited a cytologist, Robert Reeves, to join him in producing a cytogenetic study of the results. The effort ultimately led Mangelsdorf and Reeves to propose an unusual evolutionary account. Upending the more typical imagined trajectory from teosinte to maize, they theorized the hybridization of an ancestral wild maize (*Zea*) and a *Tripsacum* as the origin of teosinte. They further speculated that the hybridization of this early *Zea* and *Tripsacum* explained the distribution of maize diversity across the Americas. Other scientists, influenced by the idea that the presence of great genetic diversity indicated a region of origin for a crop species, had located the origin of domesticated corn in Mexico or Central America where variation abounded. Drawing on their cytogenetic study—supplemented with comparative morphology and other published resources that promised to shed light on maize’s past such as archaeological discoveries, anthropological accounts, and colonial-era natural histories—Mangelsdorf and Reeves mapped out a wholly different biogeography. In their account the ancestral maize had arisen and been domesticated in South America and then crossed with *Tripsacum* once it reached cultivation in Central America, giving rise to teosinte. The proliferation of varieties well known to exist in Guatemala and Mexico could be explained by imagining that teosinte (descended from the *Zea–Tripsacum* cross) had then repeatedly backcrossed with *Zea* to produce still other forms.^8^

**Figure 1:**
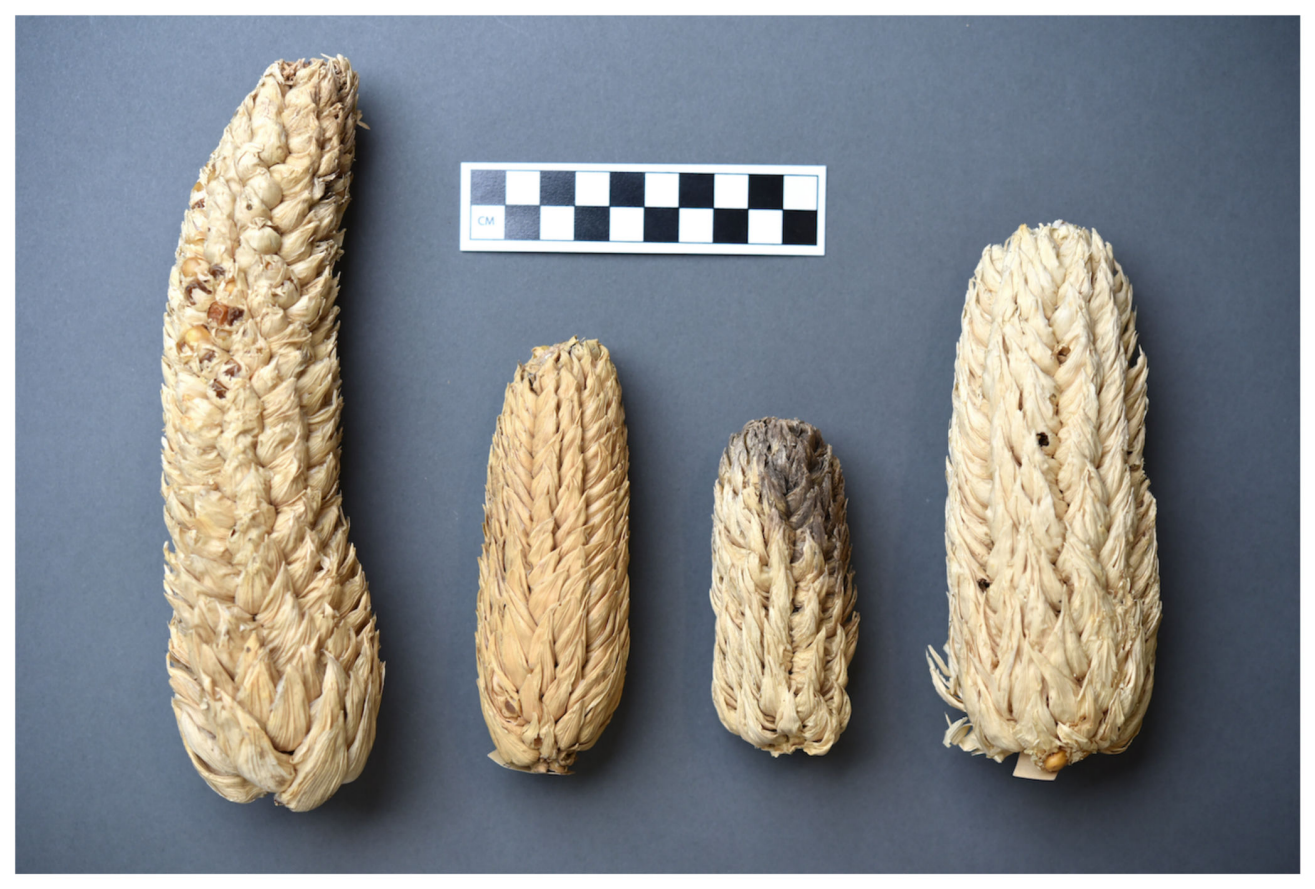
The curious pod corn (also described as “tunicate” or *Zea mays var. tunicata*), with its kernel enclosed in distinct husks, fascinated many maize researchers. These examples of pod corn were grown in Indiana in 1936 by Paul Weatherwax who, like Paul Mangelsdorf, was interested in the origins of corn. UMMAA 15466, Pod Corn, collected 1936, Bloomington, Indiana. Courtesy of the University of Michigan Museum of Anthropological Archaeology.

If this sounds involved, it was. Mangelsdorf and Reeves’ evolutionary account relied on significant conjectural conceptual work that stitched together their experimental data with a remarkably heterogeneous array of material and cultural resources on maize generated by other scholars. Not surprisingly it was immediately challenged. But Mangelsdorf was determined to stabilize the still shaky hypotheses with further empirical evidence, this time sourced directly from the field to serve his specific research agenda. In 1938 two items topped his research agenda. The first was gathering corn varieties from across South America so that he and Reeves could study their chromosomes and confirm the absence of *Tripsacum* influence. In 1938, when they first published their hypothesis, they had studied only one example of corn from South America, a striking fact given the incredible variation in maize seen across the continent. The second key research task was discovering the ancestral wild corn. Because their theory dismissed teosinte as a “recent development” in the maize family tree, Mangelsdorf and Reeves were free to return to a century-old idea in considering what the progenitor wild maize might have been. Pursuing an idea advanced in the nineteenth century by the French naturalist Etienne Geoffroy Saint-Hilaire, they proposed “pod corn”—an unusual type of maize in which each individual kernel is encased in a husk—as the putative ancestral plant. (Figure 1.) Having abandoned Mexico or Central America as the likely site of maize’s domestication they focused instead on South America. Although the diversity of maize in Peru suggested it as an important site of domestication, Peru struck Mangelsdorf and Reeves as a geographically and climatically inhospitable place for a maize-like plant to have got its start. Surely, they reasoned, the lowland savannahs of Brazil, Bolivia, and Paraguay were more favorable locales for a grassy proto-*Zea* to have lurched upward into the sunshine.^9^

This enterprising theory of the origin of domesticated maize—and, more specifically, Mangelsdorf’s determination to shore it up in the face of serious rebuttal—underwrote the Cutlers’ 1941 steamship journey to South America. Hugh Cutler had two assignments. First, he had to collect and send to Cambridge as many samples of farmer’s varieties of maize from South America as possible so that Mangelsdorf could examine these for further evidence that would support his ideas about the evolution of maize and its relatives. Second, he had to locate a living wild maize plant as incontrovertible proof that *Zea mays* had not evolved from teosinte. Were Cutler to succeed at both, Mangelsdorf and Reeves’ surprising new story of maize’s past and present just might stand.

## Tracking wild maize

At his desk in Texas, Mangelsdorf had conjectured wild maize through the examination of chromosomes with Reeves, with added inspiration from plant morphology, ancient corn cobs, stone carvings, and European natural histories. On the ground in South America, Hugh and Marian Cutler would have to rely on other approaches if they wanted to bring forth a more substantive wild maize for study. Turning conjecture into confirmation demanded Hugh’s training in ethnobotany and his experience in plant exploration. It involved engagement with unfamiliar crops, territories, and cultures, encounters that Hugh interpreted not only through his training as an ethnobotanical explorer but also the still uncertain story of maize’s past to which Mangelsdorf was committed. As a result, the evidence he returned to Mangelsdorf from the field was conditioned by the evolutionary conjectures that had propelled him there in the first place.^10^

**Figure 2:**
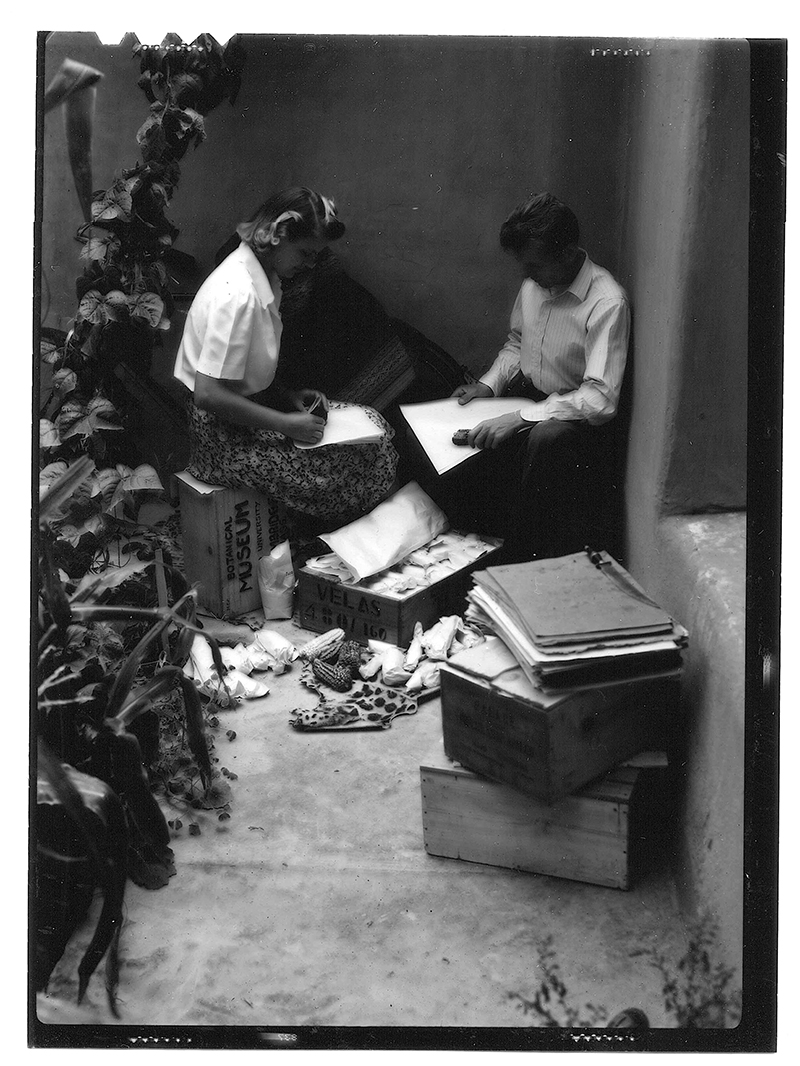
Hugh and Marian Cutler pack boxes with maize samples to send from Bolivia to Paul Mangelsdorf at Harvard in 1942, with their herbarium sheets (front right) and experimental corn (front left) in easy reach. Private collection of Bill and Elisabeth Cutler. Used by permission.

In 1940, Mangelsdorf had moved from the Texas Agricultural Experiment Station to become professor of economic botany at Harvard University. After arriving in Cambridge, he quickly raised the cash needed to send Hugh and Marian Cutler to South America in search of the maize varieties his research now demanded: both wild maize and samples of farmers’ varieties that Mangelsdorf usually called “primitive types” or “native corn.” These phrases picked out types that Mangeldorf and other scientists thought to have been in cultivation on the continent well before the arrival of “modern” varieties created by professional breeders. On paper, mention of the quest for wild maize was usually omitted, presumably to safeguard Mangelsdorf’s potential priority in a find. Instead, Hugh Cutler’s stated mission was to generate a collection of “primitive” or “native” South American maize varieties that would eventually grace a display at the Harvard Botanical Museum. The adventurous young ethnobotanist was well up to the task, especially with Marian as a collaborator. (Figure 2.) The previous year, fresh from finishing his PhD on the medicinal plant ephedra at Washington University in St. Louis, Hugh had embarked on a honeymoon trip with Marian to Mexico and Guatemala and returned with some 400 ears of “native” corn collected from local farms and markets, and many samples of *Tripsacum* and teosinte besides. Though not a botanist by formal training, Marian learned from Hugh many of the skills that plant exploration demanded, becoming a knowledgeable assistant and, at times, a research collaborator.^11^

In South America, the Cutlers’ search for maize and its relatives was shaped first by Mangelsdorf’s predictions and expectations. His theory of *Zea*’s origins circumscribed the Cutlers’ travels to a specific geographical space; it also imagined the rare settings in which a wild maize might still be clinging to existence. He and Reeves had been cautious in their expectations, writing in 1939 that “[p]erhaps the possibilities of finding wild corn even in the lowlands are not too great.” Wild corn “probably was already restricted in its habitat when man arrived on the scene” and human harvesting would have further diminished its numbers. In the intervening millennia, conditions for wild maize would have only worsened. “The introduction of cattle and other grazing animals by the Europeans has not improved the plant's chances of survival, for most herbivorous domestic animals prefer the succulent growing maize plant to almost any other vegetation,” Mangelsdorf and Reeves observed. They were therefore “reasonably certain that maize is nowhere growing in great profusion as a wild plant” but nonetheless maintained that “there is at least a remote possibility that we may yet discover, in protected sites, in the still unexplored lowlands… small colonies of the wild pod corn from which our modern maize has descended.” In other words, anyone hoping to find wild maize needed to get away from cities and corn fields and grazing animals and enter the “still unexplored” interiors of the South American continent.^12^

In 1939 Mangelsdorf already had some evidence from the field to sustain his hopes. That June he wrote to his brother Albert, who was also a geneticist and plant breeder, about a specimen of corn he had just received from a collector in Paraguay. Some months before, Mangelsdorf had sent the Austrian-born, Paraguay-based naturalist Francisco Schade, better known for his collections of birds and studies of invertebrates, a description of “what the wild [maize] plant would probably look like” and promised Schade $200 if he found it. The specimen Schade eventually returned to Mangelsdorf was, in the latter's assessment, “altogether different from any kind of corn which we have ever seen.” Unfortunately, it had no ripe seeds that Mangelsdorf could plant in hopes of multiplying the specimen for further study.^13^

**Figure 3:**
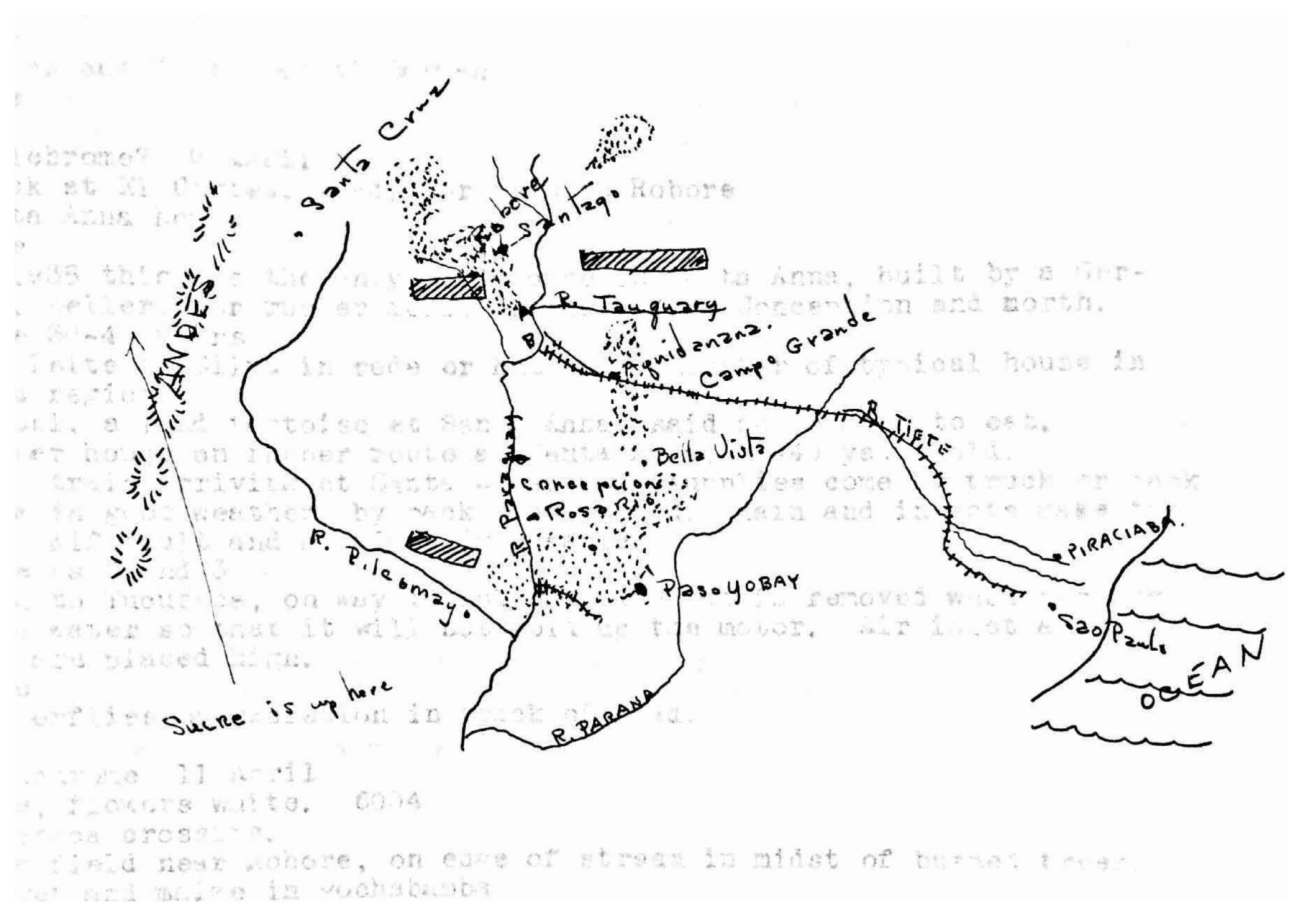
Hugh Cutler sketched a map of his journey for Paul Mangelsdorf, highlighting locations where he had secured samples of maize and other plants. There was no other way for Mangelsdorf to track his colleague’s movement or sort the samples he received in the mail. H. Cutler to P. Mangelsdorf, 24 August 1942, copy in Mangelsdorf Papers, shelf 37.8, box 3, folder: Cutler, Hugh. Harvard University Archives. Used by permission.

Two years later, one of Hugh Cutler’s early tasks in South America was to follow up on this lead. After spending an initial two and a half mostly fruitless months collecting in Brazil, he and Marian set off for Paraguay in January 1942. In Villarrica, he met up with Schade and arranged for Schade’s son, also a collector, to take him to the spot where the unusual corn sent to Mangelsdorf had been found. Traveling by horse to the town of Paso Yobai, Cutler and Schade’s son journeyed into the forest beyond, “damp with tree-ferns, bamboo, lianas, mosses” where progress was “difficult except on these trails of the tapir, deer, ant-bears & Indians.” Their destination was an opening off a main trail, which was discovered to be devoid of any traces of maize, leading Cutler to declare the original find unlikely to have been a wild corn. (Figure 3.) “[I]t is likely that Indians stopped here to put on their clothes or to eat before going down to Paso Yobay [sic],” wrote Cutler. He was skeptical about the wildness of the forest as well, believing that “there probably is no virgin forest here, that the agricultural practice of the Indians has changed all the region.” He arrived home empty-handed and exhausted after a reported twenty hours of walking and riding “without intermission.”^14^

As these comments suggest, Cutler was often more convinced of the “wildness” of the Indigenous peoples he encountered than either the maize plants or the landscapes. He regularly reported to Mangelsdorf with descriptions of Indigenous peoples, ranging from rumors of those who “walk like gorillas… have hairy foreheads, use poisoned, short arrows and are said to be cannibals” to his own first-hand assessments, for example of Guarani people he met as “gentle, polite, kind & stupid.” These observations were not idle asides about the groups he met or imagined he might encounter. Like other researchers interested in the history and taxonomy of cultivated plants, Mangelsdorf and Cutler prized local varieties that they understood to have been cultivated long before “improved” varieties had entered circulation. The ideal targets of collecting were therefore Indigenous communities, especially remote ones, where farmers were thought to cultivate varieties that had been grown by generations of their ancestors and imagined to rarely participate in commercial exchange.^15^

Thanks to researchers’ tendency to associate “primitive” corn, with its abilities to reveal the historical evolution of the species, and “primitive” peoples, who remained as relics of a distant human past, Cutler’s broader maize collecting task demanded that he acquire information about—and from—Indigenous peoples. “One of the Dept. of Ag. of Brazil men said primitive people near 10 S. Lat 65° Long. raised corn of several types but I can secure no other information as to kinds,” Cutler reported to Mangelsdorf shortly after his arrival in October 1941. A few weeks later he had an account from a “fellow in the seed section of the Dept. of Agriculture” who “said a friend of his had some pod corn from Mato Grosso Indians, no locality given.” Still in Brazil at the time, Cutler had been frustrated by his lack of access to these and other Indigenous farmers, their corn varieties, and their knowledge. Wealthy plantation owners bored him with handshakes, drinks, and idle talk and, when they finally took him to the fields as he wanted, it was inevitably “a rushed trip” with “no opportunity to talk at length with the Indians.” To get anywhere in his pursuit of “native” corn varieties he had to “go out alone to some Indian fields.”^16^

The desire to do more of this seems to have been important to the Cutlers’ decision to move on from Brazil after a few months. So, too, was the inauspicious geography and prevailing modes of agricultural production they encountered in southwestern Brazil. While based in the municipality of Corumba in December 1941 the Cutlers had “walked out … in all possible directions” and “visited places nearby so that we are certain Corumba could not now have any primitive maize.” The weather seemed to oscillate between dry and flooded in the lowlands, and the hills were “well grazed and in most places not a suitable habitat for wild corn.” Hugh speculated that he and Marian’s “trouble so far has been cattle and it will be necessary to go where they are fewer or they cannot go.” Because the imagined ancestral maize had not had to contend with cattle, which only arrived after Europeans did, its modern-day descendants presumably had no defenses against them. Unfortunately, cattle seemed ubiquitous, and even farmers who maintained “native” maize might also be raising *Bos taurus*. “Even unknown tribes, as the Chavantes [Xavante] who killed off a group of Government men several weeks ago, have some cattle,” Cutler observed to Mangelsdorf. As months went by, finding a relic plant under changed and changeable conditions of South American farms and societies must have seemed increasingly daunting, even for a skilled ethnobotanical explorer. The Cutlers nonetheless pressed on.^17^

## Cultivating connections

“Both of us are well, although at times a bit disgusted because we can’t walk out and find wild maize at once,” Hugh admitted a year later, in December 1942. Luckily looking for wild maize was not all the Cutlers had to occupy them. There were still many farmers’ varieties to collect. In addition, the scientific and social economies in which collecting was embedded entailed frequent exchanges of ideas and materials with colleagues in the United States, spurred new collaborations with scholars in Brazil and Bolivia, and rewarded activities like preparing herbarium sheets and growing specimens for study. Being an effective plant explorer and taxonomist in the 1940s required that Hugh participate in these professional exchanges, as had been true for multiple generations of his predecessors in botanical collecting and systematics. It also required that he engage collaborators often overlooked in early heroic accounts of the charting of world flora, in this case not only local and often Indigenous people whose knowledge of maize was central to his successes, but also his wife Marian, who helped him collect and press specimens, take photographs, prepare microscope slides, raise experimental plants, and more.^18^

After the trip to Paraguay in January 1942, Marian had spent several months based in Piracicaba, Brazil while Hugh traveled alone through Bolivia. Her cultivation of corn from seeds of farmers’ varieties they collected in the first few months of the journey, supplemented by the preparation of herbarium samples of corn seedlings, generated resources for the further study of diversity in maize varieties from different locations. Once Hugh returned, they worked together in preparing slides of material taken from their collection, which was now not just growing in extent but also growing as living specimens. Marian described “embedding the material in paraffin, cutting with the microtome, staining the slides + mounting, and making microscopic analyses.” As she explained to her mother, it was “the same work that [Edgar] Anderson is doing in St. Louis + Mangelsdorf at Harvard,” including in some cases with corn that she and Hugh had collected. In other words, the seeds and ears they located in farmers’ fields and local markets were multiply generative, giving rise to immediate and deferred observations, which might be about genes or cells or leaf morphology or some other feature, both on the ground in South America and back in the United States.^19^

In growing corn varieties while still based in South America, rather than on their return to the United States, the Cutlers aimed to avoid the challenges associated with growing varieties adapted to tropical climates in more temperate regions. Differences in weather and especially daylength made it difficult to transform a single ear taken from a farmers’ corn crib into several progeny ears that could be far more definitively described. The Cutlers needed these further samples to reveal patterns of inheritance—”how much the progeny varies and in what directions”—often, but not always, to resolve taxonomic questions.^20^

Working with the botanist and geneticist Edgar Anderson of the Missouri Botanical Garden, Hugh had already embarked on a classificatory project centered on defining and identifying “races” of maize. This taxonomic enterprise was related to but distinct from Mangelsdorf’s effort to tease out corn’s ancient ancestor through the study of its present-day forms. Anderson and Cutler instead wanted to build a family tree of the many diverse types of domesticated maize grown by farmers in different parts of the Americas. This tree would be built around “races,” which they defined as distinct subpopulations of the species *Zea mays*. A race was “a group of related individuals with enough characteristics in common to permit their recognition as a group.” They explained this concept chiefly by comparison to human “races,” since “in both cases it is not easy to work out the racial composition of the whole and it is difficult to give a precise definition to the term ‘race.’” Despite the acknowledged impossibility of definitively bounding freely interbreeding populations, Anderson and Cutler considered a “natural” classification of maize varieties, based on genetically distinctive “races” and organized according to evolutionary relatedness, to be an essential tool for understanding the history of the species and its cultivation. Given the analogy between human races and maize races, Cutler and Anderson leaned on human race science in their methods, for example following the approach of physical anthropologist Earnest Hooton in grouping subpopulations by “general perception” of their similarities (Hooton grouped skulls, they grouped ears of corn), then measuring and calculating averages for each subgroup. A final step involved checking these confirmed subgroups against “recognized geographical and ethnic barriers”—the moment where the relationship between human and maize populations might have been acknowledged as more than analogic.^21^

**Figure 4:**
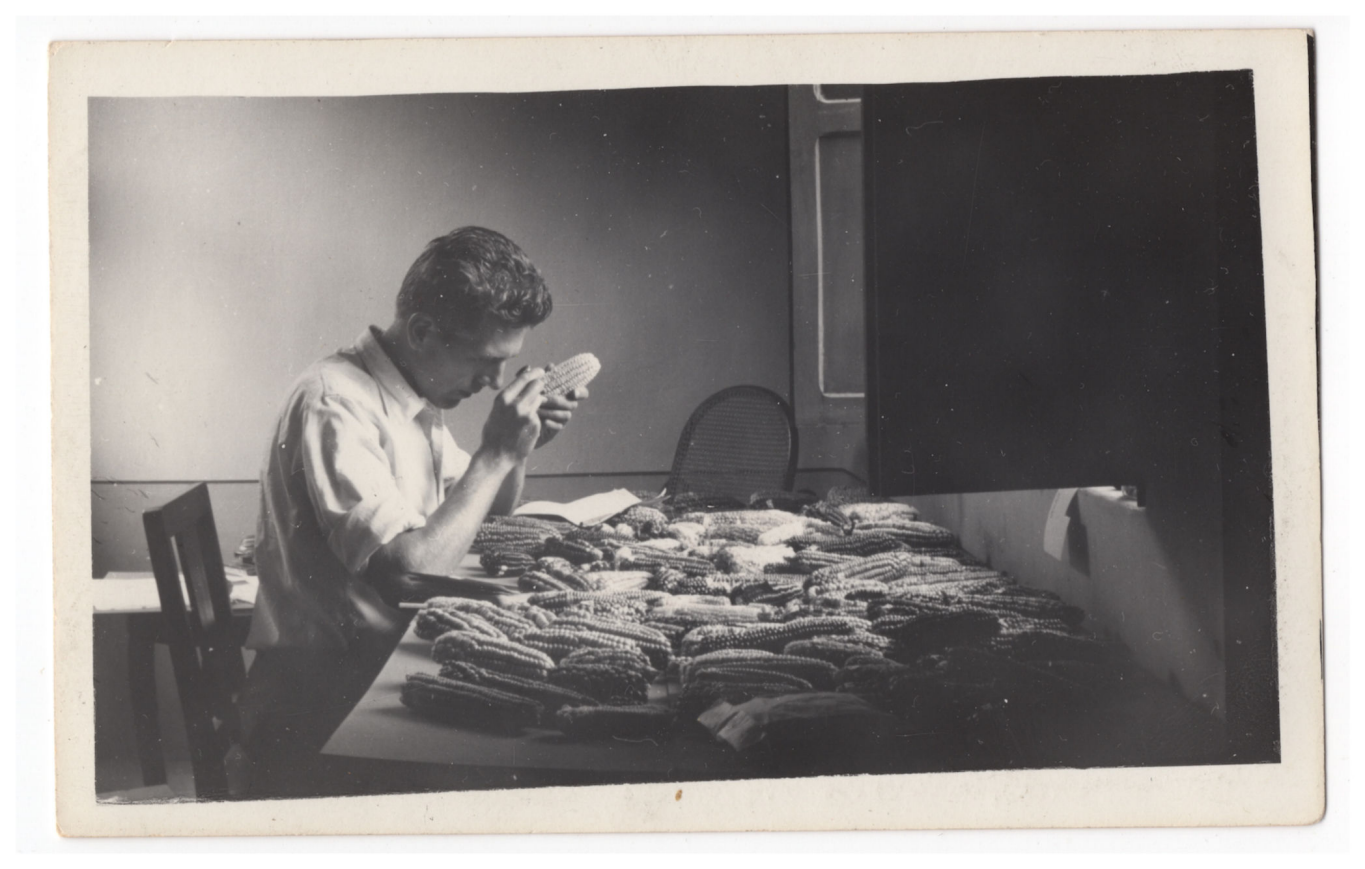
Hugh Cutler examining highland maize varieties while in Bolivia in 1942. Private collection of Bill and Elisabeth Cutler. Used by permission.

**Figure 5:**
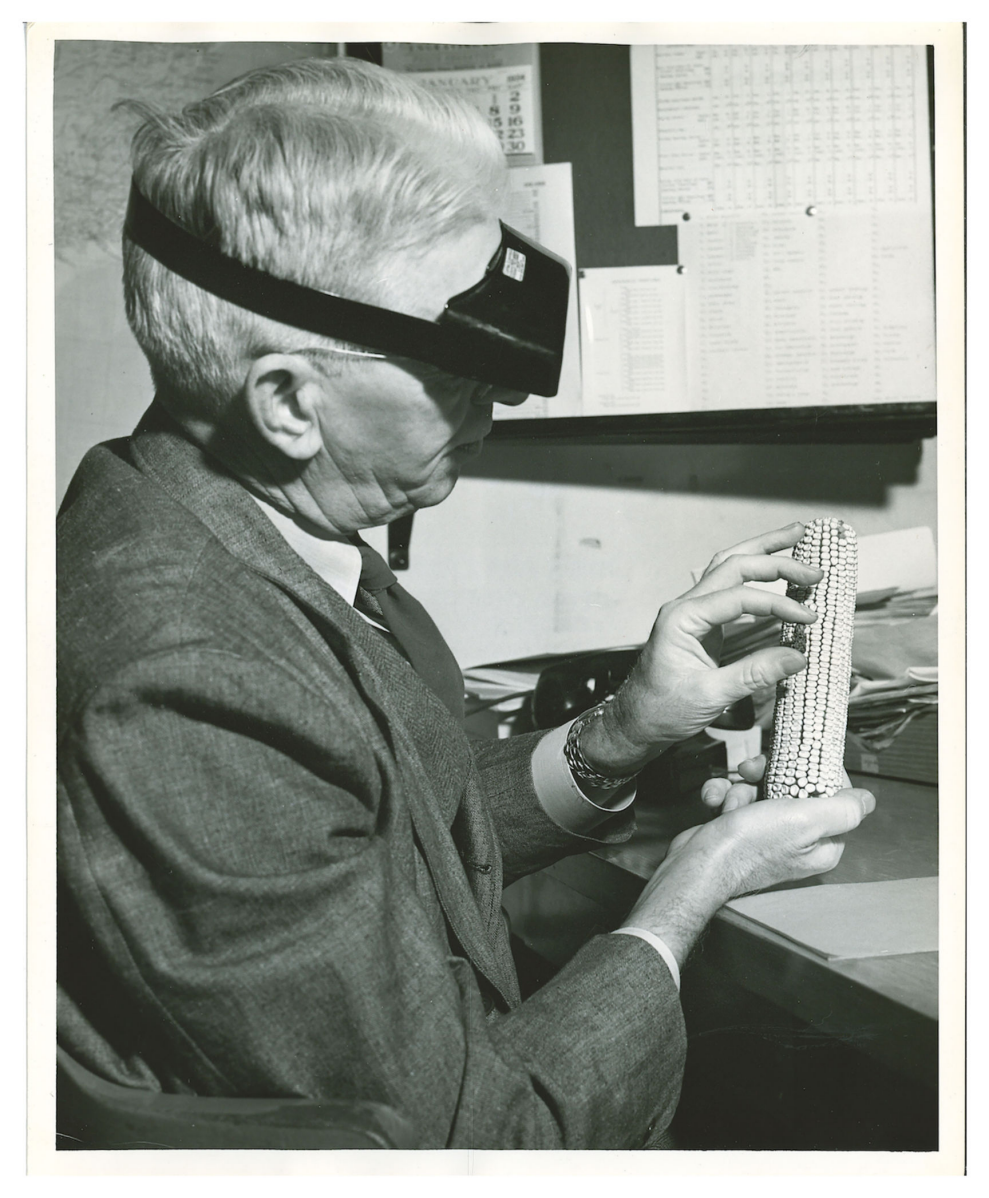
Paul Mangelsdorf, sporting combination eyeshade and low-powered magnifying lenses, compares a tiny “primitive corn cob” from a New Mexico archaeological site to a “modern” maize variety. Photo by Harvard University News Office, 1954. HUP Mangelsdorf, Paul C. (10). Harvard University Archives. Used by permission.

Cutler and Anderson’s production of taxonomies of domesticated maize depended especially on having samples of distinct varieties. (Figure 4.) These materials were typically gathered on journeys like Hugh and Marian’s and could be measured and compared once back at a desk or a lab, and in some cases cultivated, crossed, and cultivated again. (Figure 5.) For example, in their first study of maize races, Cutler and Anderson considered varieties from the southwestern United States, Mexico, and Guatemala. For southwestern US maize, they relied on about fifty samples gathered by Cutler and the geographer George Carter. For Mexico and Guatemala, they had the materials obtained by the Cutlers on their honeymoon trip plus samples shared by an additional three researchers. In some cases, they worked with seeds and ears that had come straight from the field, while in others these materials came from a second- or later-generation plant grown from field collected seeds. Cutler and Anderson emphasized that the samples produced by growing out seed collected earlier had been “grown in triplicate at St. Louis, Mo., College Station, Texas, and Cienfuegos, Cuba,” a triangulation meant to reveal physical changes in the maize plant induced by changing environmental circumstances as opposed to reliable hereditary features. This, too, motivated Hugh and Marian to cultivate the samples they needed for later study while based in South America: plants that stayed in place might prove more stable specimens for study.^22^

Not that taxonomy was the sole impetus for their efforts. Finding new diversity in a crop as central to American economies as corn was a tantalizing prospect. By establishing a maize patch of South American farmers’ varieties under Marian’s care (with the help of local “laborers and assistants” for the hoeing, weeding, watering, and so on), Hugh hoped not only to “find some of value in [his] studies” but also “something spectacular” that would be “of particular value in producing quick-growing, high-yield varieties for the [United] [S]tates.” He and Marian kickstarted the process by crossing some of their South American samples with US varieties, hoping to locate types that mixed well standard lines of the US corn belt. In one shipment of seed to Mangelsdorf, he advised that the senior scientist pass along these crosses to “some one who will be interested in using it.” He specifically recommended the “Palta Waltaco (or paltal hualtacu),” a large-grained floury maize whose kernels were typically peeled for eating as *mote*, whole nixtamalized corn grains (a preparation also known as hominy).^23^

Hugh Cutler had first learned the name and uses of Palta Waltaco/paltal hualtacu from the Bolivian scientist Martín Cárdenas, a botanist who had quickly become Cutler’s key contact in that country and eventually proved the most influential of several South American researchers on Cutler’s developing knowledge of South American maize. When he and Marian had first landed in Brazil, Cutler had exchanged information with Carlos Krug, an agronomist and geneticist, and the German émigré geneticist Freidrich Brieger. Both were based at institutions in the Brazilian state of São Paulo and had corn collections and knowledge to share. Mangelsdorf was eager for Cutler to obtain anything he could by way of samples from either scientist. On arrival, Cutler deemed Krug’s contributions distinctly inferior. Although he had a large collection of maize, it had been collected by someone else, and “practically all… from markets” rather than directly from Indigenous farmers. Brieger, by comparison, had “more information on corn than anyone so far” including seeds and samples of “primitive” maize gathered from various Indigenous communities in the region. Still, Cutler did not send it on immediately as Mangelsdorf wanted because he felt it had been poorly maintained. “All of it is much crossed,” Cutler explained, and it was “necessary to go back to [Brieger’s] lists and check them all to find which had been mixed with imported corn.”^24^

Although he dismissed most of Krug and Brieger’s maize samples a couple of items stood out to Cutler as valuable: each scientist had examples of pod corn reportedly sourced from somewhere in São Paulo. These ears immediately suggested to Cutler that “tunicate”—another name for pod corn—”is apparently quite common.” Here was the evidence needed to embolden a hunt for a pod corn–like wild maize of the kind imagined by Mangelsdorf. However, Cutler eventually judged these samples as unhelpful from the perspective of identifying ancestral maize, just as he had dismissed Krug and Brieger’s larger maize collections as resources for understanding and taxonomizing “native” South American maize. As Cutler explained to Mangelsdorf, Krug and Brieger’s pod corns from São Paulo likely reflected the mixing of commercial corns from elsewhere in the world, especially from the US and Europe, with local strains; in France, for example, pod corns had sometimes been sold as novelties, making it possible for their genes to mix with other varieties in cultivation and subsequently spread. It was only Martín Cárdenas who had a pod corn reportedly obtained directly from “an Indian source.”^25^

## Maize and medicine men

Cárdenas gamely joined Cutler on the search for further exemplars of pod corn, obtained directly from the field or market, in the region around Cochabamba, Bolivia, in 1942. This stop on Hugh Cutler’s journey would spark his own new narrative of corn’s past. His ambitious account of pod corn linked the diverse incoming evidence he confronted—from Mangelsdorf’s evolutionary hypotheses to Cárdenas’s expertise on Bolivian maize, from Indigenous herbalists’ rare wares in Cochabamba to his earlier ethnobotanical experiences in the American West—to generate a novel narrative of maize on the move through time, space, and culture.

Before he set foot in Bolivia, Cutler had reason to believe the region would harbor a rich diversity in maize types he sought for Mangelsdorf’s collection, if not the wild maize plant itself, thanks to descriptions he’d received from Friedrich Brieger and others. This supposition was immediately confirmed. In an initial set of notes, he established the main types of Bolivia according to Cárdenas, ten varieties identified by local name, basic characteristics, and principal uses. Palatal hualtacu was listed as being “for mote [hominy] and harina [flour]” with “[g]rains flat, yellow, flour” and “plants occasionally more vigourous than Huillcaparo, ears large.” Huillcaparo had dark brown grains and a rust-colored cob and was mainly used for flour and in chicha (fermented maize beer). The “small plants, small ears” of Pisankalla could be round or pointed, and in either case would “explode on toasting.” And so on. To the information supplied by Cárdenas, Cutler appended further names and uses gleaned from local farmers, for example an “Indian near San Benito” told him alternative names for Cárdenas’s Huillcaparo and Uchuquilla. He also continued learning Quechua names for different maize dishes—and which of these to savor—as he scoured markets and fields for examples of distinct Bolivian varieties.^26^

By May, Cutler had 80 ears from Bolivia ready to ship to Mangelsdorf, and he reckoned on getting about 400 more before leaving the country. Further samples of pod corn proved elusive, however. “Pod corn is hard to locate,” he explained to Mangelsdorf in May 1942, “and altho I have looked at hundreds & more of granaries & in many more fields & stores & many markets, I have found none.” He had assiduously followed up on two different reports of wild maize plants called Totáchi growing near Robore, only to discover ordinary sorghum. However, locals seemed to know about pod corn, calling it by the name *paca sara* (elsewhere transcribed as *puca sara*) or “hidden corn” according to Cutler, and he made a point of visiting “herb stores” because he knew some Indigenous people to keep “odd shaped ears … as charms.” He soon learned that in Bolivia a different kind of maize, *cuti sara* or “turned corn” was considered useful in treating a range of illnesses and began asking after this as well. The *cuti sara* maize was described by Cutler has having “two grains in a spikelet,” a physiological variation that produced striking kernel patterns. “I have asked several hundred Indians,” he reported, “venders, farmers, cargeadores [porters], etc.” Still, *cuti sara* was also “quite rare.”^27^

Cutler finally located both pod corn and “turned corn” while visiting a market with Martín Cárdenas, amid what Cutler described as “the stock-in-trade of one of those wandering cure vendors” of Bolivia. Cutler associated the vendor with a specific cultural group and practice, namely the “Callahuayo [sic] Indians… travelling medicine men… taught from birth to know the virtues of many plants” who sojourned not only across the Andean highlands but all of the continent and beyond, “carrying their packs of remedies.” This vendor had in his possession “an old and battered ear of pod corn with some grains missing” and an ear of turned corn with its unusual double-grained spikelet. As Cutler remembered the encounter, the “medicine seller was quite drunk” and “passed out shortly afterwards, and he refused to sell the ears.” Cutler, undeterred by these events, “took them [the ears] and gave him what a bystander said was the price.” He evidently could not countenance leaving such prized research objects behind.^28^

**Figure 6:**
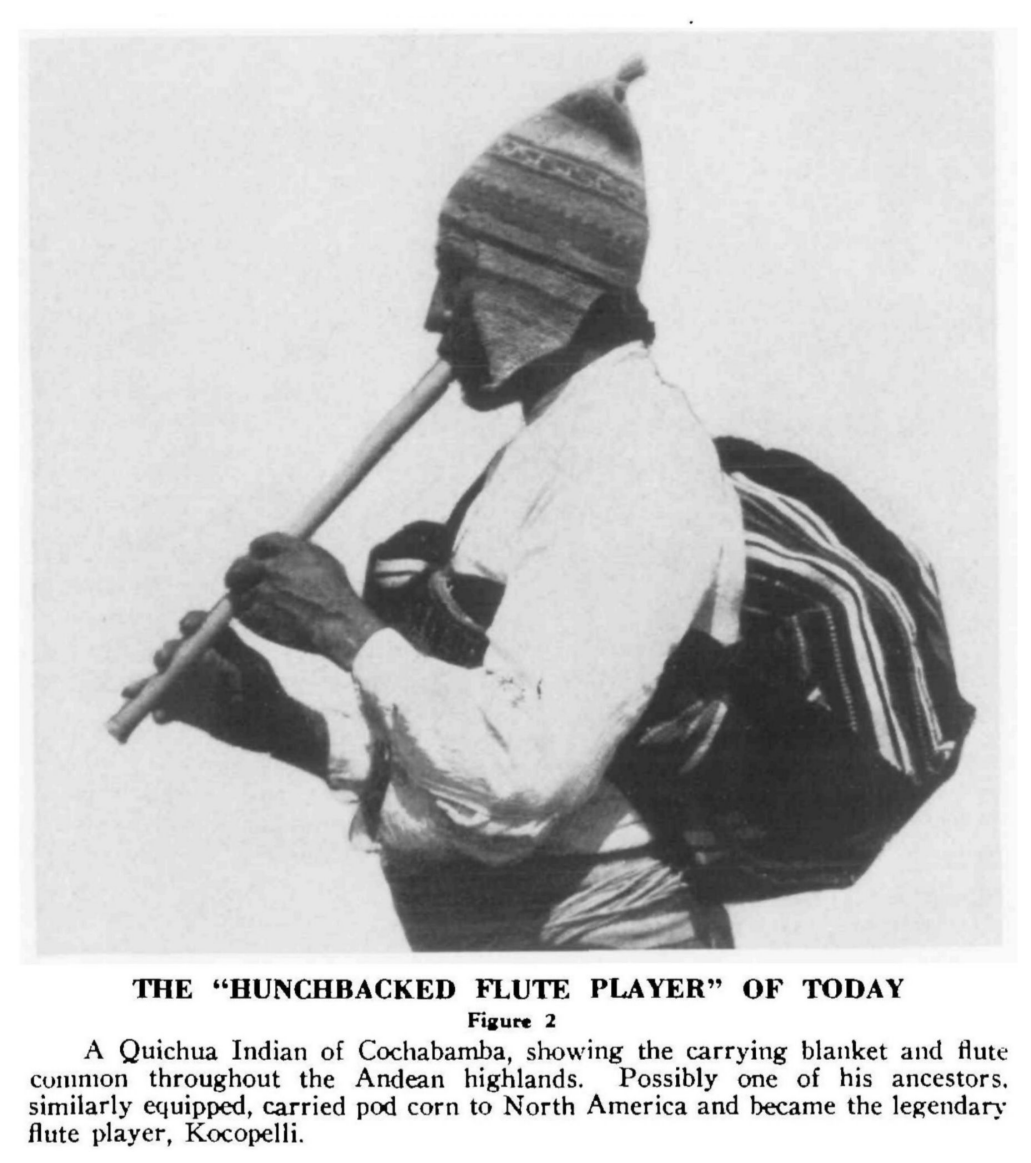
Drawing on evidence from field samples of maize, anthropological accounts, and archeological artefacts, Hugh Cutler hypothesized in the 1940s that South American healers carrying curative plants could explain the survival and spread of pod corn. Hugh C. Cutler, “Medicine Men and the Preservation of a Relict Gene in Maize,” *Journal of Heredity* 35 (1944), 291. [Permission to be sought.]

The experience proved influential to Cutler’s theorizing. Pod corn and turned corn had both been ascribed curative properties in some South American traditions, which meant they were exemplars of maize’s diverse cultural as well as biological forms. This opened the door for novel ideas about the evolutionary history of maize, inflected now with ethnobotanical knowledge. In Cutler’s assessment, the existence of pod corn seemingly defied understanding, given that its key feature, husked kernels, would have been selected against in even the most straightforward of efforts to keep good corn seed for future cultivation. Yet rather than attribute pod corn to a recent and likely repeated mutation that had been perpetuated as a novelty, Cutler floated the possibility that pod corn traced back to an ancient, albeit still unknown, ancestor. The traveling medicine men of the Andean highlands were known to play flutes. (Figure 6.) According to Cutler, who’d done some of his earliest collecting in the southwestern desert of the United States, “The flute, the pack of remedies slung over the back, and the long trips remind one of the rock pictures of the ‘hunchbacked flute player’ occasionally called Kocopelli [sic]” sometimes seen in that region. Cutler also knew of a petrified ear of maize found in Arizona that could plausibly be assessed as a pod corn. If one stood back and connected these dots, it seemed possible that “the ancient Southwestern hunchback was actually a Callahuayo medicine man bringing to North America the character of tunicate maize [pod corn] which had not been introduced earlier in ears selected for food use.”^29^

This evolutionary tale, published in a short report for the *Journal of Heredity* and illustrated with images of both the maize and men involved in the story, was one of a handful of scientific publications Hugh Cutler produced after he and Marian brought their foraging and farming abroad to an end. (Their return was delayed until 1945; their corn work was put aside while Hugh sought rubber sources for the war effort and Marian was employed by the US state department to run a cultural institute in Brazil.) The pod corn–Kokopelli paper was perhaps the closest thing to an account of wild maize that Cutler was able to produce after years of searching. It was not the clear documentation of a surviving ancient plant that Mangelsdorf had hoped for at the outset, but instead an intensely speculative recreation of a pathway for survival of a single “relict gene.” A definitive story of maize’s past lie in the future.

## Accounting for absence

Without a wild maize in hand, neither Hugh Cutler nor Mangelsdorf could provide a convincing story about the origins of maize based on the Cutlers' exploration of South American maize. It proved easier to float theories about why a wild maize couldn't be located. They pointed to the conversion of “wild” land to pastures for cattle, along with historic and contemporary changes in agricultural practice, as reasons why a wild maize would be rare or even extinct. A follow-up journey to South America by Hugh Cutler in 1948 added another possibility to the list. Hired by Mangelsdorf for “another quick look for wild corn,” Cutler arrived in Bolivia in early 1947 only to be stymied at every turn by heavy rains and flooding. “The rain has slowed everything up & made many trails impassable as well as rivers,” Cutler reported from the field. “Besides, some of the pampa and savannas are under water & there is no food.” Although the rain prevented Mangelsdorf from receiving the range of maize samples he’d anticipated, it proved to be a piece of evidence in and of itself. “If wild corn were by any chance a plant indigenous to savannas which are now subject to violent flooding perhaps it has indeed disappeared,” he proposed to Cutler, invoking the ur-extinction narrative of Western cultures as a novel explanation for the absence of a hypothesized plant.^30^

**Figure 7:**
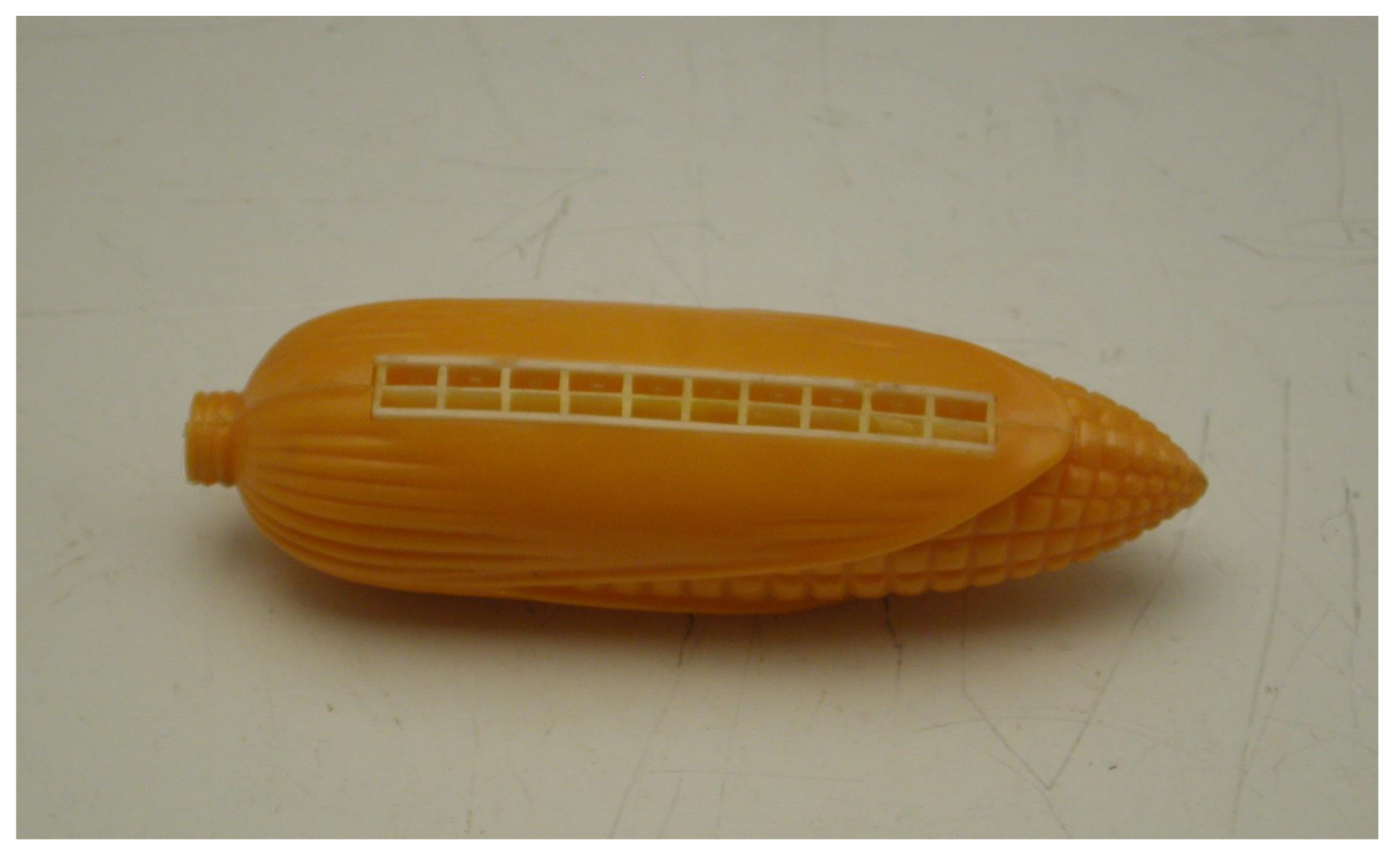
A plastic harmonica made in Hong Kong from Paul and Peg Mangelsdorf’s corn object collection. Accession no. 1981.006.014, plastic corn harmonica, unknown maker, Hong Kong, c. 1965. Gregg Musuem of Art & Design, Gift of Dr. and Mrs. Paul C. Mangelsdorf. Used by permission.

Wild maize apparently missed the ark, but Hugh Cutler survived the floods of 1947 and returned to a position he had secured as curator of economic botany at the Field Museum of Natural History in Chicago. Expanding his research into archaeology and paleobotany, and moving to the Missouri Botanical Garden in 1953, he maintained a focus on the useful plants of the Americas. He ultimately amassed his own collection of more than 12,000 maize ears for ethnobotanical study. Paul Mangelsdorf remained animated by maize, too, in particular the question of its origins. In the decades that followed, he continued to advocate for his and Reeves’ theory of the origin of maize, responding energetically to other scientists’ efforts to refocus attention on teosinte as the progenitor of domesticated maize. Although the collection of maize varieties he established at Harvard disintegrated in storage, a companion collection of maize objects and artefacts survives. According to a recent description, this assemblage reflects decades of effort by Mangelsdorf and his wife Peg to obtain “anything and everything to do with corn”: corn-shaped dishes and cutlery, ivory carvings and ceramic figures, and novelties including “Christmas tree lights, a harmonica, a tie clip, and even a corn-shaped straight razor.” With objects sourced from around the world, the collection captures a wider range of maize’s unique forms—in most cases domestic, rather than domesticated—than Mangelsdorf’s herbarium specimens ever could. (Figure 7.)^31^

In the twenty-first century, many details of maize’s past are still under investigation. And many will surely remain so. As this essay has shown, the obscure past of a ubiquitous crop generated opportunities for researchers to assemble and reassemble scattered shards of evidence into potentially meaningful mosaics. By charting the contours of one peculiar plant exploration mission—the Cutlers’ quest for manifold maize varieties and a speculative wild plant with untold unspeculative domesticated descendants—rather than a succession of evolutionary theories, the history documented here offers a chance to reflect on the kinds of evidence that sustained agricultural taxonomy. This evidence included collections of seeds and plants, along with slides of cells and tissues, observations of fields and farms, and maps of climate and topography. It also included encounters with and stories about peoples and cultures. This was not always appreciated by researchers. While in Bolivia, Hugh Cutler found himself reminding Mangelsdorf, “Corn spread is probably so tied up with migration that races and battles will have to be studied more than botany.” He might have added that accounts of the origin and spread of maize would also be tied up with his own history, and that of researchers like him, individuals who inevitably embedded their own experiences and expectations into the evolutionary stories they spun.^32^

## Footnotes

1 Marian Cutler, diary, 6 and 7 October 1941, private collection of Bill and Elisabeth Cutler (hereafter Cutler collection); Rueben Goossens, “S.S. Uruguay, Brazil, and Argentina: Page Two,” available at http://ssmaritime.com/SS-Uruguay-Brazil-Argentina-2.htm, accessed 5 September 2022.

2 P. C. Mangelsdorf and R. G. Reeves, "The Origin of Indian Corn and Its Relatives," *Bulletin of the Texas Agricultural Experiment Station* 574 (1939), 7–315, on 250; P. Mangelsdorf to C. A. Krug, 28 October 1941, Papers of Paul C. Mangelsdorf, Harvard University Archives (hereafter Mangelsdorf Papers), shelf 37.6, box 1, folder: Cutler, Hugh.

3 On state-led collection and use of crop diversity in breeding, see Christophe Bonneuil, “Seeing Nature as a ‘Universal Store of Genes’: How Biological Diversity Became ‘Genetic Resources’, 1890–1940.” *Studies in History and Philosophy of Science Part C* 75 (2019), 1–14; Marianna Fenzi and Christophe Bonneuil, “From ‘Genetic Resources’ to ‘Ecosystems Services’: A Century of Science and Global Policies for Crop Diversity Conservation,” *Culture, Agriculture, Food and Environment* 38 (2016), 72–83; Tiago Saraiva, “Breeding Europe: Crop Diversity, Gene Banks, and Commoners,” in Nil Disco and Eda Kranakis (eds.), *Cosmopolitan Commons: Sharing Resources and Risks Across Borders* (Cambridge, MA, 2013), 185–212; Michael Flitner, “Genetic Geographies: A Historical Comparison of Agrarian Modernization and Eugenic Thought in Germany, the Soviet Union, and the United States,” *Geoforum* 34 (2003), 175–185; Olga Elina, Susanne Heim, and Nils Roll-Hansen, “Plant Breeding on the Front: Imperialism, War, and Exploitation,” *Osiris* 20 (2005), 161–179. On twentieth-century biological specimen collections, see Mary E. Sunderland, “Specimens and Collections,” in Bernard Lightman (ed.), *A Companion to the History of Science* (Chichester, UK: 2016), 488–99; Bruno J. Strasser, “Collecting Nature: Practices, Styles, and Narratives,” *Osiris* 27 (2012): 303–340. For accounts involving the history of crop plant taxonomy, see Kim Kleinman, “His Own Synthesis: Corn, Edgar Anderson, and Evolutionary Theory in the 1940s,” *Journal of the History of Biology* 32 (1999), 293–320; Diana Alejandra Méndez Rojas, “Los Libros del Maíz. Revolución Verde y diversidad biológica en América Latina, 1951–1970,” *Letras Históricas* 24 (2021), 149–182; Helen Anne Curry, “Taxonomy, Race Science, and Mexican Maize,” *Isis* 112 (2021), 1–21. An account that emphasizes crop plant exploration as a taxonomic enterprise is Fabian Kremer, Kärin Nickelsen, and Dana von Suffrin, “Botany and the Science of History: Nature, Culture, and the Origins of Civilization, circa 1850–1900,” *Isis* 113 (2022), 45–62.

4 Helen Anne Curry, *Endangered Maize: Industrial Agriculture and the Crisis of Extinction* (Oakland, CA, 2022), chapter 2; P. Mangelsdorf to H. Cutler, 23 March 1944, Mangelsdorf Papers, shelf 37.8, box 3, Folder: Cutler, Hugh.

5 This study is inspired by and indebted to Harriet Ritvo’s magnificent and groundbreaking treatment of taxonomy and culture, *The Platypus and the Mermaid, and Other Figments of the Classifying Imagination* (Cambridge, MA, 1998).

6 Paul C. Mangelsdorf, *Corn: Its Origin, Evolution and Improvement* (Cambridge, MA, 1974), vii–viii; James A. Birchler, “Paul C. Mangelsdorf, 1899–1989,” *Biographical Memoirs of the National Academy of Sciences* (Washington, DC, 2014). For an overview of debates on the origin of maize, see John Doebley, “George Beadle’s Other Hypothesis: One-Gene, One-Trait,” *Genetics* 158 (2001), 487–493. On the history of evolutionary studies of maize with a focus on the desk-based tools used in this research, see Kleinman, “His Own Synthesis”; Kim Kleinman, “Edgar Anderson: Interdisciplinary Authority on What Was *Not* Known about Corn,” *Endeavour* 23 (1999), 114–117.

7 The gamut of theories is reviewed in P. C. Mangelsdorf and R. G. Reeves, "The Origin of Maize," *Proceedings of the National Academy of Sciences* 24 (1938), 303–312, on 303–304. See also Paul Weatherwax, “The Phylogeny of Zea Mays,” *The American Midland Naturalist* 16 (1935), 1–71. For a more recent resolution of the question, see John Doebley, “The Genetics of Maize Evolution,” *Annual Review of Genetics* 38 (2004), 37–59.

8 Mangelsdorf and Reeves, “The Origin of Maize”; Mangelsdorf and Reeves, “The Origin of Indian Corn and Its Relatives”; S. M. Bukasov, “The Cultivated Plants of Mexico, Guatemala and Columbia,” *Bulletin of Applied Botany, Genetics, and Plant Breeding* [Leningrad], supplement 47 (1930), English summary on 470–553. On crop taxonomy as an interdisciplinary endeavor along the lines pursued by Mangelsdorf, see Kleinman, “His Own Synthesis”; Kleinman, “Edgar Anderson.”

9 G. W. Beadle, “Teosinte and the Origin of Maize,” *Journal of Heredity* 30 (1939), 245–247; Mangelsdorf and Reeves, “The Origin of Maize”; Mangelsdorf and Reeves, “The Origin of Indian Corn and Its Relatives,” 309–311. On George Beadle’s objections to Mangelsdorf and Reeves’ proposal, see Doebley, “George Beadle’s Other Hypothesis.”

10 On plant exploration and studies of crop plant evolution, see Bonneuil, “Seeing Nature as a ‘Universal Store of Genes.’” The Russian geneticist Nikolai Vavilov looms large in this history; further accounts of Vavilov that emphasize the connections between his plant exploration and studies of crop evolution include Elena Aronova, *Scientific History: Experiments in History and Politics from the Bolshevik Revolution to the End of the Cold War* (Chicago and London, 2021), ch 3; Igor G. Loskutov, *Vavilov and His Institute: A History of the World Collection of Plant Genetic Resources in Russia* (Rome, 1999).

11 E. Anderson to P. Mangelsdorf, 17 December 1940, Mangelsdorf Papers, shelf 37.8, box 1, folder: Anderson, Edgar (2 of 2); David Browman, "Necrology: Hugh Carson Cutler," *Bulletin of the History of Archaeology* 9 (1999): 1–6.

12 Mangelsdorf and Reeves, “The Origin of Indian Corn and Its Relatives,” 250–251.

13 P. Mangelsdorf to A. J. Mangelsdorf, 20 June 1939, Mangelsdorf Papers, shelf 37.6, box 1, folder: Correspondence 1939; Floyd Edward Hayes, “Status, Distribution and Biogeography of the Birds of Paraguay,” PhD dissertation, Loma Linda University, 1993, 71–72.

14 H. Cutler to P. Mangelsdorf, 28 December 1941, Mangelsdorf Papers, shelf 37.8, box 3, folder: Cutler, Hugh; H. Cutler to P. Mangelsdorf, 10 January 1942, Mangelsdorf Papers, shelf 37.8, box 3, folder: Cutler, Hugh; H. Cutler to P. Mangelsdorf, 24 January 1942, Mangelsdorf Papers, shelf 37.8, box 3, folder: Cutler, Hugh; Marian Cutler, diary, 22 January 1948, Cutler collection.

15 H. Cutler to P. Mangelsdorf, 10 January 1942, Mangelsdorf Papers, shelf 37.8, box 3, folder: Cutler, Hugh; Cutler to Mangelsdorf, 9 February 1942, shelf 37.8, box 3, folder: Cutler, Hugh; P. Mangelsdorf to M. Jenkins, 13 May 1940, Mangelsdorf Papers, shelf 37.6, box 1, folder: Jenkins, Merle T. On twentieth-century assumptions about “primitive” crop varieties in relation to cultural change, see Bonneuil, “Seeing Nature as a ‘Universal Store of Genes’”; Stephen B. Brush, *Farmers’ Bounty: Locating Crop Diversity in the Contemporary World* (New Haven, 2004).

16 H. Cutler to P. Mangelsdorf, 16 October 1941, Mangelsdorf Papers, shelf 37.8, box 3, folder: Cutler, Hugh; H. Cutler to P. Mangelsdorf, 15 November 1941, Mangelsdorf Papers, shelf 37.8, box 3, folder: Cutler, Hugh; H. Cutler to P. Mangelsdorf, 25 December 1941, Mangelsdorf Papers, shelf 37.8, box 3, folder: Cutler, Hugh.

17 M. and H. Cutler to P. Mangelsdorf, 9 December 1941, Mangelsdorf Papers, shelf 37.8, box 3, folder: Cutler, Hugh; H. Cutler to P. Mangelsdorf, 21 December 1941, Mangelsdorf Papers, shelf 37.8, box 3, folder: Cutler, Hugh.

18 On the economies of botanical collections in different social and political contexts, see, e.g., E. C. Spary, *Utopia’s Garden: French Natural History from Old Regime to Revolution* (Chicago, 2000); Jim Endersby, “A Garden Enclosed: Botanical Barter in Sydney, 1818–39,” *British Journal for the History of Science* 33 (2000), 313–334; Staffan Müller-Wille, “Nature as a Marketplace: The Political Economy of Linnaean Botany,” *History of Political Economy* 35 (2003), 154–172; Sarah Easterby-Smith, *Cultivating Commerce: Cultures of Botany in Britain and France, 1760–1815* (Cambridge, 2018). On the reliance of European and American plant exploration on local expertise and labor across different periods and contexts, see, e.g., Londa Schiebinger, *Plants and Empire: Colonial Bioprospecting in the Atlantic World* (Cambridge, MA, 2004); Kapil Raj, *Relocating Modern Science: Circulation and the Construction of Knowledge in South Asia and Europe, 1650–1900* (Basingstoke: Palgrave Macmillan, 2007); Kathleen S. Murphy, “Translating the Vernacular: Indigenous and African Knowledge in the Eighteenth-Century British Atlantic,” *Atlantic Studies* 8 (2011), 29–48; Kalle Kananoja, *Healing Knowledge in Atlantic Africa: Medical Encounters, 1500–1850* (Cambridge, UK, 2021). There is also a significant literature on women’s participation in plant exploration; for a useful overview (and twentieth century example), see Nuala C. Johnson, “On the Colonial Frontier: Gender, Exploration and Plant-hunting on Mount Victoria in Early 20^th^-century Burma,” *Transactions of the Institute of British Geographers* 42 (2017), 417–31. For an introduction to invisible labor more generally, see Jenny Bangham, Xan Chacko, and Judith Kaplan, *Invisible Labour in Modern Science* (London, 2022).

19 H. Cutler to P. Mangelsdorf, 8 December 1942, Mangelsdorf Papers, shelf 37.8, box 3, folder: Cutler, Hugh; M. Cutler to “Mother”, 25 July 1942, Cutler collection.

20 H. Cutler to “Dad”, 28 March 1942, Cutler collection.

21 Edgar Anderson and Hugh Cutler, “Races of Zea Mays: I. Their Recognition and Classification,” *Annals of the Missouri Botanical Garden* 29 (1942), 69–88, on 71, 77. On the history of research into races of maize, see Curry, “Taxonomy, Race Science, and Mexican Maize”; Méndez Rojas, “Los Libros del Maíz.”

22 Anderson and Cutler, “Races of Zea Mays,” 77, 79.

23 H. Cutler to “Dad”, 28 March 1942, Cutler collection; H. Cutler to P. Mangelsdorf, 19 January 194[3], Mangelsdorf Papers, shelf 37.6, box 1, folder: Cutler, Hugh.

24 M. and H. Cutler to P. Mangelsdorf, 27 November 1941, Mangelsdorf Papers, shelf 37.8, Box 3, Folder: Cutler, Hugh. For biographical information on these scientists, see H. F. Linskens, “Friedrich Gustav Brieger, 1900–1985,” *Berichte der Deutsche Botanische Gesellschaft* 99 (1986), 137–143; “Krug, Carlos Arnaldo,” Dicionário Histórico-Biográfico das Ciências da Saúde no Brasil (1832–1930), https://dichistoriasaude.coc.fiocruz.br/iah/pt/verbetes/krug.htm (accessed 14 November 2022); Mario Rodríguez Rodríguez, *Martín Cardenas: El Eximio Botánico y Naturalista de América* (La Paz, 2005). See also Francisco M. Salano, “The Evolution of Science in a Latin-American Country: Genetics and Genomics in Brazil,” *Genetics* 208 (2018), 823–832.

25 M. and H. Cutler to P. Mangelsdorf, 27 November 1941, Mangelsdorf Papers, shelf 37.8, Box 3, Folder: Cutler, Hugh; H. Cutler to P. Mangelsdorf, 24 August 1942, Mangelsdorf Papers, shelf 37.8, box 3, folder: Cutler, Hugh.

26 H. Cutler, “Corn Types According to Dr. Cardenas,” 16 June 1942, Mangelsdorf Papers, shelf 37.8, box 3, folder: Cutler, Hugh.

27 H. Cutler to P. Mangelsdorf, 3 May 1942, Mangelsdorf Papers, shelf 37.8, box 3, folder: Cutler, Hugh; H. Cutler to P. Mangelsdorf, 2 April 1942, Mangelsdorf Papers, shelf 37.8, box 3, folder: Cutler, Hugh; H. Cutler, “Corn Types According to Dr. Cardenas,” 20 May 1942, Mangelsdorf Papers, shelf 37.8, box 3, folder: Cutler, Hugh; H. Cutler to P. Mangelsdorf, 15 May 1942, Mangelsdorf Papers, shelf 37.8, box 3, folder: Cutler, Hugh; H. Cutler to P. Mangelsdorf, 24 August 1942, Mangelsdorf Papers, shelf 37.8, box 3, folder: Cutler, Hugh.

28 Hugh C. Cutler, “Medicine Men and the Preservation of a Relict Gene in Maize,” *Journal of Heredity* 35 (1944), 291–294, on 293; H. Cutler to P. Mangelsdorf, 4 May 1948, Mangelsdorf Papers, shelf 37.8, box 2, folder: Cutler, Hugh 1 of 2.

29 H. Cutler to P. Mangelsdorf, 24 August 1942, Mangelsdorf Papers, shelf 37.8, box 3, folder: Cutler, Hugh; Cutler, "Medicine Men and the Preservation of a Relict Gene in Maize," 293–294.

30 H. Cutler to P. Mangelsdorf, 27 December 1945, Mangelsdorf Papers, shelf 37.8, box 2, folder: Cutler, Hugh 2 of 2; H. Cutler to P. Mangelsdorf, 20 March 20 [1947], Mangelsdorf Papers, shelf 37.8, box 2, folder: Cutler, Hugh 1 of 2; P. Mangelsdorf to H. Cutler, 4 April 1947, Mangelsdorf Papers, shelf 37.8, box 2, folder: Cutler, Hugh 1 of 2.

31 Browman, “Necrology: Hugh Carson Cutler,” 2–3; Mangelsdorf, *Corn*; Birchler, “Paul C. Mangelsdorf,” 4; Gregg Museum of Art and Design, “Paul Mangelsdorf: An Un-corn-ventional Collector,” online exhibit, http://searchgreggcollection.arts.ncsu.edu/mExhibit.aspx?rID=Web.2021.005&dir=GALLERY%20OF%20ART# (accessed 28 September 2022). For the standard twenty-first century account of maize’s origins, see Doebley, “The Genetics of Maize Evolution.”

32 H. Cutler to P. Mangelsdorf, 19 January 1942, Mangelsdorf Papers, shelf 37.6, box 1, folder: Cutler, Hugh. For recent studies of maize evolution and domestication, see, e.g., Michelle C. Stitzer and Jeffrey Ross-Ibarra, “Maize Domestication and Gene Interaction,” *New Phytol*ogist 220 (218), 395–408; Logan Kistler, Heather B. Thakar, Amber M. VanDerwarker, Alejandra Domic, Anders Bergström, Richard J. George, Thomas K. Harper, Robin G. Allaby, Kenneth Hirth, and Douglas J. Kennett, “Archaeological Central American Maize Genomes Suggest Ancient Gene Flow from South America,” *Proceedings of the National Academy of Sciences* 117 (2020), 33124–33129; Abraham-Juárez, María Jazmín, Allison C. Barnes, Alejandro Aragón-Raygoza, Destiny Tyson, Andi Kur, Josh Strable, and Rubén Rellán-Álvarez, “The Arches and Spandrels of Maize Domestication, Adaptation, and Improvement,” *Current Opinion in Plant Biology*, Cell biology, 64 (2021), 102124.

